# Measuring the Value of Research Data: A Citation Analysis of Oceanographic Data Sets

**DOI:** 10.1371/journal.pone.0092590

**Published:** 2014-03-26

**Authors:** Christopher W. Belter

**Affiliations:** LAC Group, Central Library, National Oceanic and Atmospheric Administration, Silver Spring, Maryland, United States of America; Institute of Marine Research, Norway

## Abstract

Evaluation of scientific research is becoming increasingly reliant on publication-based bibliometric indicators, which may result in the devaluation of other scientific activities - such as data curation – that do not necessarily result in the production of scientific publications. This issue may undermine the movement to openly share and cite data sets in scientific publications because researchers are unlikely to devote the effort necessary to curate their research data if they are unlikely to receive credit for doing so. This analysis attempts to demonstrate the bibliometric impact of properly curated and openly accessible data sets by attempting to generate citation counts for three data sets archived at the National Oceanographic Data Center. My findings suggest that all three data sets are highly cited, with estimated citation counts in most cases higher than 99% of all the journal articles published in Oceanography during the same years. I also find that methods of citing and referring to these data sets in scientific publications are highly inconsistent, despite the fact that a formal citation format is suggested for each data set. These findings have important implications for developing a data citation format, encouraging researchers to properly curate their research data, and evaluating the bibliometric impact of individuals and institutions.

## Introduction

In recent years there has been increasing interest in, and use of, bibliometric indicators for the evaluation and ranking of research institutions. Bibliometric indicators feature prominently in global mixed-method ranking schemes such as the Academic Ranking of World Universities [1] and the Times Higher Education ranking [2]. They also feature in national mixed-method research assessment exercises in the UK, Brussels, Italy, and Australia. Other global ranking schemes are based solely on bibliometric indicators [3], [4]. Bibliometric indicators are often recommended to supplement [5]–[9] or even replace [10], [11] peer review in evaluating research institutions.

Partially in response to the growing importance of bibliometrics in research evaluation, and partially in response to other factors, there is also a growing movement focused on the development of a standard method of citing data sets in academic publications [12]. Reasons for developing a citation format for data sets include verification of published results, reuse of data sets for additional research purposes, and attribution to data collectors and archivists. Such suggestions have been made in bioinformatics [13]–[16], genetics [17], climate sciences [18], geochemistry [19]–[21], oceanography [22], [23], earth sciences [24], [25], and multidisciplinary sciences [26]–[28], among others.

Although there is widespread agreement within the movement that a minimum set of information is necessary to a complete data set citation, there seems to be two schools of thought as to how this ought to be accomplished. One school favors a direct citation to the data set as it resides in an established repository. This model was first adopted for nucleotide sequence datasets in the formation of GenBank [29] and adapted for the marine [30] and earth [31], [32] sciences before being more widely recommended [33]–[35] and implemented in various subject-specific and general data repositories such as the California Digital Library (http://www.cdlib.org/), DataONE (http://www.dataone.org/), the Dataverse Network (http://thedata.org/), Dryad (http://datadryad.org/), ICPSR (http://www.icpsr.umich.edu/), Pangaea (http://www.pangaea.de/), NOAA's climatic (http://www.ncdc.noaa.gov/), geophysical (http://www.ngdc.noaa.gov/), and oceanographic (http://www.nodc.noaa.gov/) data centers, etc. One of the fundamental components of this model is the creation and citation of an identifier that uniquely identifies the data set being cited. This identifier typically takes the form of a doi assigned through DataCite [36], although other identifiers may also be used.

This model is beginning to be incorporated into the products of commercial scientific information providers. In 2012, Thomson Reuters launched the Data Citation Index (http://wokinfo.com/products_tools/multidisciplinary/dci/), a database of data sets that provides suggested citation formats for each data set indexed in the database and attempts to generate citation linkages to articles indexed in its other Web of Science databases. More recently, Elsevier, in cooperation with DataCite and numerous data repositories, launched a similar project that attempts to link papers available in ScienceDirect to the data sets that they use or have deposited in repositories (http://www.elsevier.com/about/content-innovation/database-linking) through data set dois or other unique identifiers.

The other school of thought favors the citation of a ‘data paper' or ‘data publication’ describing the data set. In this model, the metadata necessary for using a data set and a link to the data set is presented in a paper published either in a traditional journal or in a specialized data journal. Data papers differ from more traditional publications in that no analyses or findings resulting from the data set are required. Researchers wishing to cite the data set would then cite the data paper, rather than the data set. This model has been suggested in the neurosciences [37], [38], genetic sciences [39], and bioinformatics [40] communities, and implemented in the geosciences community through the formation of data journals such as *Earth System Science Data* (http://www.earth-system-science-data.net) and *Geoscience Data Journal* (http://onlinelibrary.wiley.com/journal/10.1002/%28ISSN%292049-6060) and the publication of data papers in journals such as the *Quarterly Journal of the Royal Meteorological Society, Eos*, and *Oceanography*. Examples of recent data papers in the earth sciences include those describing the ERA-40 reanalysis in atmospheric sciences [41], the Argo profiling floats [42], and a database of iron enrichment experiment results [43] in oceanography.

Closely tied to the development of data citation standards is the growing awareness of the need to properly preserve, describe, and provide access to data sets, a collection of activities sometimes referred to as data curation. In order for a data set to be cited, it must first have been deposited in a repository, preserved in an interoperable format, adequately described by a formal set of metadata attached to the data set, and made available to other researchers for reuse. Although technical issues exist at each step in this process, the idea of sharing data sets with other researchers has proven to be the most controversial [44]–[47]. In addition to concerns over the idea of freely sharing research data, many researchers are reluctant to devote the time necessary to properly curate their research data, especially since many have not received training on how to do so. Although mandates for data preservation and sharing have been established by the National Science Foundation (http://www.nsf.gov/bfa/dias/policy/dmp.jsp), the American Geophysical Union (http://publications.agu.org/author-resource-center/publication-policies/data-policy/), and the US Office of Science and Technology Policy (http://www.whitehouse.gov/blog/2013/02/22/expanding-public-access-results-federally-funded-research) among others, it is not yet clear whether these mandates will motivate researchers to do so in the future.

Although bibliometric indicators can be a useful compliment to peer review in the evaluation of scientific research [7]–[9], the growing reliance on publication-based indicators for research evaluation could potentially lead to the devaluation of activities that do not typically result in the publication of articles in scientific journals. Participation in workshops, policy formulation, peer-review of submitted manuscripts, public education, and mentoring are all critical to the advancement of scientific research and to the translation of that research into societal benefits, but few of these activities ‘count’ in bibliometric evaluations because they rarely result in formal publications. Since the incorporation of bibliometric indicators into research evaluation is known to affect the subsequent behavior of those being evaluated [48]–[51], it seems likely that the growing reliance on bibliometric indicators could result in a disincentive to engage in such activities.

One such activity that is likely to be devalued in this context is data curation. Despite its importance to the scientific community, data curation rarely results in the production of scientific journal articles, meaning that scientists and institutions devoting time and effort to data curation are unlikely to be rated favorably by bibliometric indicators in comparison with their more prolific peers. This is likely to undermine support for data curation efforts, since scientists are unlikely to devote the time and effort required to properly curate their data sets if they are unlikely to be rewarded for doing so.

The purpose of this analysis is to combine these trends by attempting to show the value of data curation in bibliometric terms. Specifically, I attempt to generate citation counts for three oceanographic data sets curated by the National Oceanic and Atmospheric Administration (NOAA)'s National Oceanographic Data Center (NODC). In doing so, I hope to demonstrate the utility of data curation to scientific research, since these data sets could not have been cited without the curation activities performed by NODC and its partners. In the process, I also hope to inform the discussion surrounding the development of data citation standards by identifying how these data sets are currently cited and referenced in scientific articles. Such baseline information can be useful in identifying both the metadata that should be included in a data citation format and how such a format ought to be applied.

Although many articles advocating data citation standards mention the usefulness of such standards for bibliometric evaluation, efforts to actually generate citation counts for data sets are fairly rare. Chao [52] measured dataset reuse in the earth sciences and found that earth science data sets were primarily cited in physical science and multidisciplinary journals, suggesting that data sets generated in one discipline may also have applications in other disciplines. Parsons et al. [24] used Google Scholar to search for mentions of snow cover data sets archived at the National Snow and Ice Data Center and found between 100 and 600 mentions per year. Piwowar and colleagues [53], [54] used a similar method, searching PubMed Central for mentions of data sets archived in the Gene Expression Omnibus (GEO) database and estimated that GEO data sets had been cited over 1,150 times by the end of 2010. The Inter-university Consortium for Political and Social Research (ICPSR) maintains a bibliography of several thousand publications that cite one or more data sets archived by ICPSR [55]. Finally, several studies suggest that articles with publically available data sets are more highly cited than articles that do not make their data publically available [e.g. 56,57].

## Methods

In consultation with NODC, I selected three highly-used data sets for this analysis: the World Ocean Atlas and World Ocean Database (WOA/WOD), the Pathfinder Sea Surface Temperature (PSST) data set, and the Group for High Resolution Sea Surface Temperature (GHRSST) data set. The World Ocean Atlas is a quality-controlled set of objectively analyzed global *in situ* observational data published in four volumes focused on the variables of temperature [58], salinity [59], oxygen [60], and nutrients [61]. Although NODC considers the World Ocean Atlas a data product, rather than a raw data set, because it is a compilation of many individual data sets gathered at various times and locations around the world and because of the quality control and analysis done on the underlying data, I consider it a data set for the purposes of this analysis. The World Ocean Database [62] is an interactive database of the data used to create the World Ocean Atlas. Since the Atlas and the Database utilize same underlying data, I will refer to them in combination as the WOA/WOD. The WOA/WOD was initially published in 1982 as the ‘Climatological Atlas of the World Ocean’ [63] and rereleased with updated data in 1994, 1998, 2002, 2006, and 2009–2010. The PSST data set [64] is a long-term set of global sea surface temperature data derived from the Advanced Very High Resolution Radiometer (AVHRR) sensor mounted on NOAA's polar-orbiting satellites. The GHRSST data set [65], [66] is a global set of combined satellite and in situ sea surface temperature data contributed by a number of institutions from around the world. GHRSST data are initially collected from these institutions by the NASA Jet Propulsion Laboratory and then transferred 30 days after observation to NODC for long term preservation and access.

In the first phase of this analysis, conducted in March 2013, I attempt to generate citation counts for these data sets using three data sources: Web of Science, Science Citation Index Expanded (WoS), the full text search capabilities provided by various journal publishers' websites (Elsevier, Springer, Wiley, etc), and Google Scholar. I search these data sources to find citations to, or mentions of, these data sets in scientific publications and compile the number of results retrieved. In this analysis, I count mentions of these data sets as citations, a broader definition of ‘citation’ than is currently used, because scientific articles utilizing or discussing these data sets may or may not formally cite them. This definition is consistent with that employed by previous studies [24], [53], [54]. The search strings used in this phase are listed in Table 1. These search strings are deliberately restrictive to improve the precision of the retrieved results. As a result, although the resulting counts are likely to be fairly, although not entirely, accurate, they are also likely to be undercounts of the actual number of publications citing or mentioning these data sets.

**Table 1:** Search strings used to generate citation counts for three data sets in WoS, publishers' full text websites, and Google Scholar.

Data Set	Search String
WOA/WOD	“world ocean atlas” OR “world ocean database”
PSST	“pathfinder sst” OR “pathfinder sea surface” OR “avhrr sst”
GHRSST	GHRSST OR “group for high resolution” OR “goade high resolution”

To generate citation counts for each data set in Web of Science, I use the search strings to search the title, abstract, keywords, and funding text (or acknowledgements) fields and add all of the resulting records to my ‘Marked List.’ I then perform cited reference searches to identify citations to publications or reports associated with each data set and add all of the resulting records to my ‘Marked List’. The final number of records in my ‘Marked List’ is then noted as the WoS citation count for each data set. Using the ‘Marked List’ in this way allows me to avoid potentially double counting records retrieved by multiple searches.

To generate citation counts for each data set using publishers' websites, I use the search strings to search the full text of all records on each site using the sites' internal search engine and note the number of results retrieved for each data set. I then combine these totals across websites to generate a final citation count for each data set. The publishers' sites searched were: ScienceDirect, SpringerLink, the Wiley Online Library, the American Meteorological Society's Online Journals page, the Nature Publishing Group website, the Science (AAAS) website, the PNAS website, Taylor and Francis Online, IEEE Xplore, the Public Library of Science website, the American Chemical Society website, and the Ecological Society of America's online journals site. Searching each of these sites individually was necessary because no formal full text database covering the oceanographic, marine, and geo- sciences is available.

To generate citation counts for each data set using Google Scholar, I use the search strings to search the database without restriction and then note the number of results retrieved as the final citation count. Due to known indexing and metadata issues with Google Scholar [67], [68], these counts are likely to be inflated, and include non-peer-reviewed publication types, but are also likely to provide reasonable estimates of how often these sets are used overall and to provide accurate rankings of these data sets relative to each other [69], [70].

In the second phase of this analysis, conducted in January 2014, I attempt a more comprehensive cited reference search in WoS to generate citation counts to all editions of the WOA/WOD over time. Since WOA/WOD originally was, and still is, distributed as a print publication, it seems likely that formal citations to this data set would be more numerous than for the other data sets analyzed here. To allow for wide variance in citation formats, I search for the author(s) and publication year(s) of each edition of the WOA/WOD and then manually select the relevant search results. The search strategies used in this process are summarized in Table 2.

**Table 2:** Search strategies used to identify articles citing each edition of the WOA/WOD in WoS.

Edition	Search Strategy
1982	Cited Author = (levitus s*) AND Cited Year = 1982
1994	Cited Author = (conkright m* OR levitus s* OR boyer t*) AND Cited Year = 1994
1998	Cited Author = (antonov j* OR levitus s* OR boyer t* OR conkright m*) AND Cited Year = 1998
2001	Cited Author = (stephens c* OR boyer t* OR conkright m* OR locarnini r* OR levitus s*) AND Cited Year = 2002
2005	Cited Author = (boyer t* OR locarnini r* OR antonov j* OR garcia h*) AND Cited Year = 2006
2009	Cited Author = (boyer t* OR antonov j* OR garcia h* OR locarnini r*) AND Cited Year = (2009 OR 2010)

The process of executing these search strategies for one edition of the WOA/WOD is as follows. First, I perform a Cited Reference Search using the search criteria listed in Table 2. In step 2 of the Cited Reference Search process, I select the relevant citation variants through manual inspection of the step 1 results, and manually count the number of cited reference variants selected. After retrieving the final list of citing articles, I then analyze the results using the tools provided by WoS to obtain citation counts per year, subject category, and country for that edition of the WOA/WOD. I then repeat this process for each subsequent edition of the WOA/WOD.

## Results

**Figure 1:**
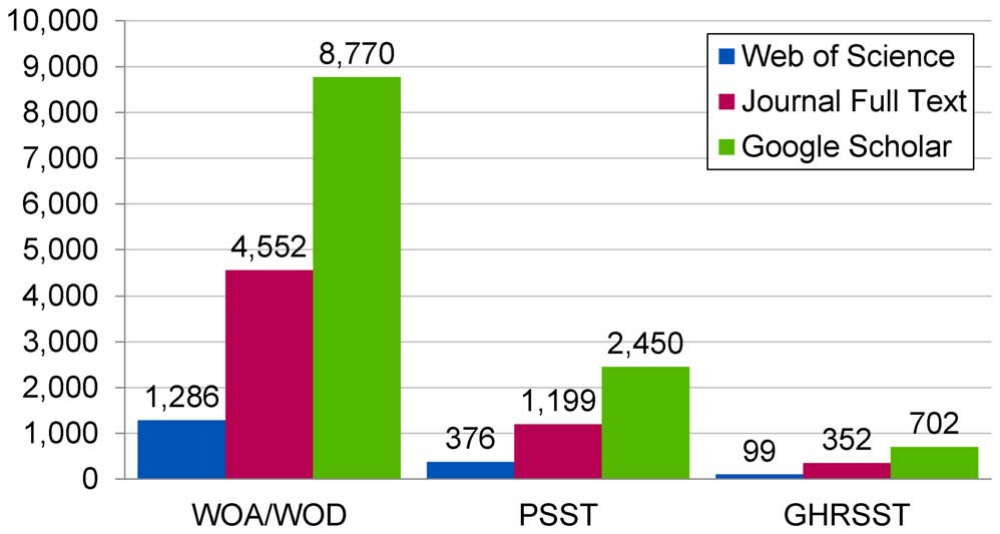
Citation counts retrieved for three oceanographic data sets from three data sources.

The citation counts generated for WOA/WOD, PSST, and GHRSST during the first phase of this analysis are summarized in Figure 1. Two consistent patterns seem to emerge in these counts. First, the total number of citations generated for each data set increases as the coverage of the data source increases. WoS is the most limited of the three data sources, since it only indexes article metadata, acknowledgements, and cited references. Publishers' sites have wider coverage, since they allow access to articles' full text, but I only searched a limited number of these sites. Google Scholar has the broadest coverage, in that it offers access to the full text of a broad range of publishers' websites as well as to conference proceedings, institutional repositories, and other websites. The citation counts generated using these data sources seem to follow this pattern, with citation counts generated from publishers' sites being nearly four times higher than those generated from WoS and counts generated from Google Scholar being nearly eight times higher than WoS.

Second, the data sets are consistently ranked relative to each other across the three data sources. The citation counts for WOA/WOD are higher than those for PSST, which are higher than those for GHRSST. The magnitude of these differences also seems consistent, with WOA/WOD receiving approximately four times more citations in each data source than PSST and PSST receiving approximately three times more citations than GHRSST. The consistency of these patterns across data sets and data sources suggests that these findings are robust, although much additional work would be necessary to verify their accuracy.

**Figure 2:**
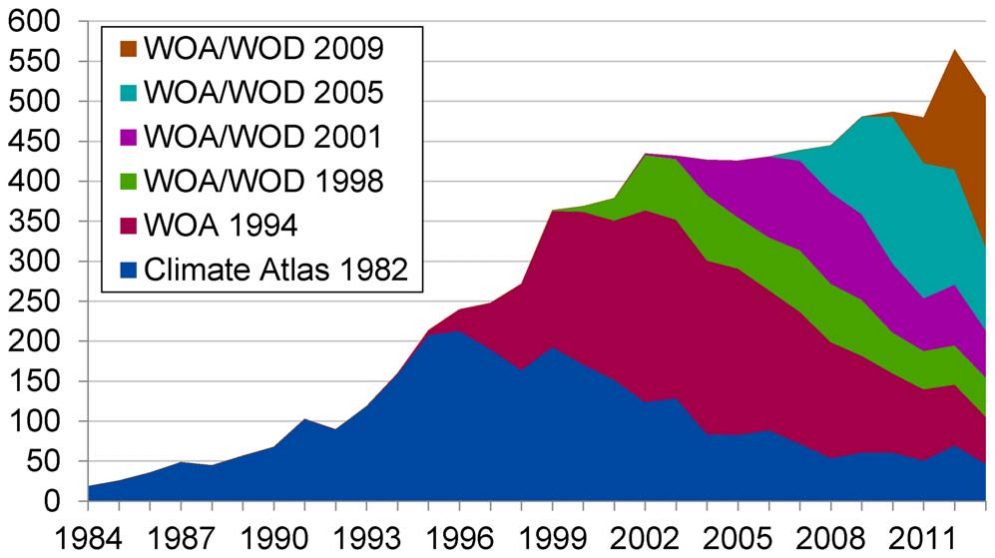
Number of citations received per year by all editions of the WOA/WOD.

In compiling these citation counts, I also find a wide variety in the methods used to refer to these data sets. Examples of this variety are given in Figure 2. Some articles include formal citations to these data sets, but the format of these citations is highly variable, despite the fact that NODC provides a suggested citation format for each of these data sets. Many other articles simply mention the data set in the text of the article, although the format of such mentions is also highly variable. The data sets are referred to by various names (PSST alone is referred to as ‘Pathfinder Sea Surface Temperature’, ‘Pathfinder SST’, ‘Advanced Very High Resolution Radiometer SST′,’ AVHRR SST′, etc) and a URL to the online source of the data is not always included.

In the second phase of this analysis, a more comprehensive Cited Reference Search in WoS for articles citing WOA/WOD, I find a total of 8,412 articles citing all six editions of the WOA/WOD from 1984 to 2013. The 1982 edition has been cited 2,987 times, the 1994 edition has been cited 2,577 times, the 1998 edition has been cited 810 times, the 2001 edition has been cited 842 times, the 2005 edition has been cited 795 times, and the 2009 edition has been cited 401 times. The distribution of articles citing WOA/WOD over editions and years is presented in Figure 2.

These distributions display a number of interesting features. First, versions of the WOA/WOD seem to require at least four, and up to 14, years after their initial release date to reach their peak citation rate. The time necessary for versions to reach their peak rate has declined with each version: the 1982 Climatological Atlas reached its peak 14 years after its initial publication, whereas the 1998 version required six years and the 2005 version required four. The amount of time necessary for the older versions of the WOA/WOD to reach their peak citation rate is longer than the 2–5 years required for most journal articles [71], [72], although the 2005 version seems to have peaked within that timeframe.

This delay may be due to the media in which each successive version of WOA/WOD was distributed. The 1982 climatological atlas was distributed via magnetic tape and personal communication, the 1994 and 1998 editions were distributed via CD-ROM, the 2001 version was distributed via DVD, the 2005 was distributed via DVD and online access, and the 2009 version was distributed online. Each successive version made the data more accessible and usable, possibly leading to quicker incorporation of the data into scientific articles. In addition, updates began to be incorporated into the WOA/WOD every three months starting in 2008, allowing the WOA/WOD to be used for more timely investigations.

Second, although all releases of the WOA/WOD are highly cited, some versions are clearly more highly cited than others. The 1982, 1994, and 2009 versions all received over 200 citations in a single year, whereas the 1998 version never received more than 82 citations in a single year and the 2001 version never received more than 114. Since the 1998 and 2001 versions presented similar data, were prepared using similar methods, and compiled by many of the same authors as the 1994 and 2009 versions, it is unclear what conclusions to draw from these trends. In addition, the 2005 version seems to have been the most rapidly cited version of the data set, accumulating 795 citations in the eight years since it was published, although the 2009 version seems to be following a similar trajectory, having received 401 citations in the five years following its publication.

Finally, all versions of the WOA/WOD continue to be highly cited well beyond their publication date, even when one or several newer versions of the data set are available. The 1982, 1994, 1998, and 2001 versions all received between 50 and 60 citations in 2013. It is unclear whether researchers continue to use the older versions of the WOA/WOD out of habit, convenience, unawareness of newer versions, or other reasons. For whatever reason, researchers continue to cite these data sets well beyond the cited article half-life of 9.1 years recorded for Oceanography in the 2011 edition of *Journal Citation Reports*, suggesting that all versions of the WOA/WOD continue to be valuable resources for scientific research.

**Figure 3:**
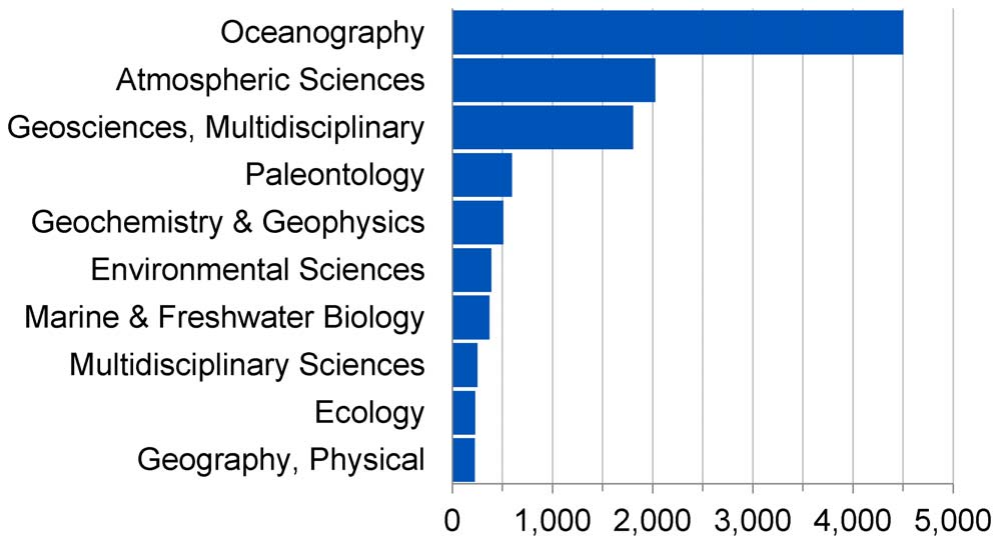
Number of articles citing all versions of the WOA/WOD per WoS-defined subject category.

Analysis of the articles citing all versions of the WOA/WOD also reveals some interesting features. An analysis of the WoS subject categories of these citing articles, presented in Figure 3, shows that although the WOA/WOD is predominantly cited by articles in Oceanography, it is also cited by other related fields. The high number of citations from the Meteorology & Atmospheric Sciences category suggests that the WOA/WOD is frequently used by studies examining the effects of the ocean on weather and climate. Its number of citations in Paleontology, primarily by articles published in the journal *Paleoceanography*, suggests that it is used in studies of the prehistoric ocean as well as those of the modern ocean. Finally, its use in the Environmental Sciences, Marine and Freshwater Biology, and Ecology subject categories suggest that it is being used by studies examining the effects of ocean conditions on marine biota.

**Figure 4:**
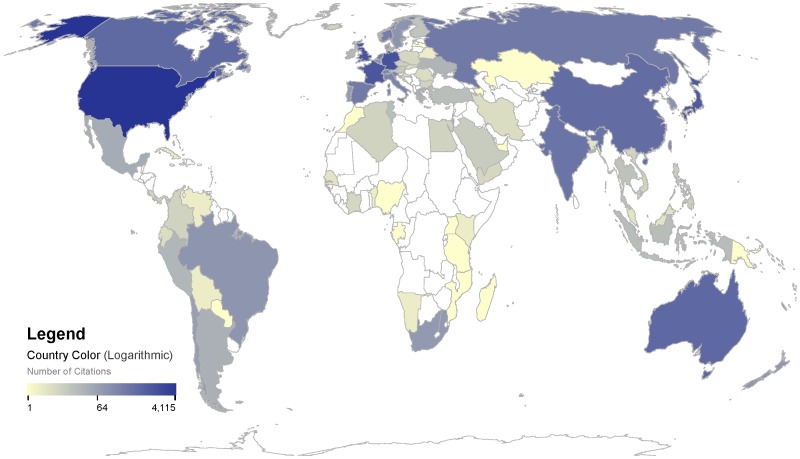
Number of articles citing all versions of the WOA/WOD per country.

Figure 4 presents an analysis of these citing articles by country. Citing articles by authors from multiple countries are counted as whole citations for each country, rather than fractionally. Creation of the WOA/WOD is an international project in that the WOA/WOD consists of data sets contributed by researchers from numerous countries around the world. Figure 4 suggests that the international nature of its creation is reflected by the international scope of its use. The WOA/WOD is not only highly cited by the traditionally prolific scientific countries such as the United States, France, Germany, and the United Kingdom, but also by rising scientific countries such as China, India, and Brazil. This suggests that although NODC is an institution of the US government, its work to archive, quality control, and freely provide the data comprising the WOA/WOD is useful to the global scientific community.

**Figure 5:**
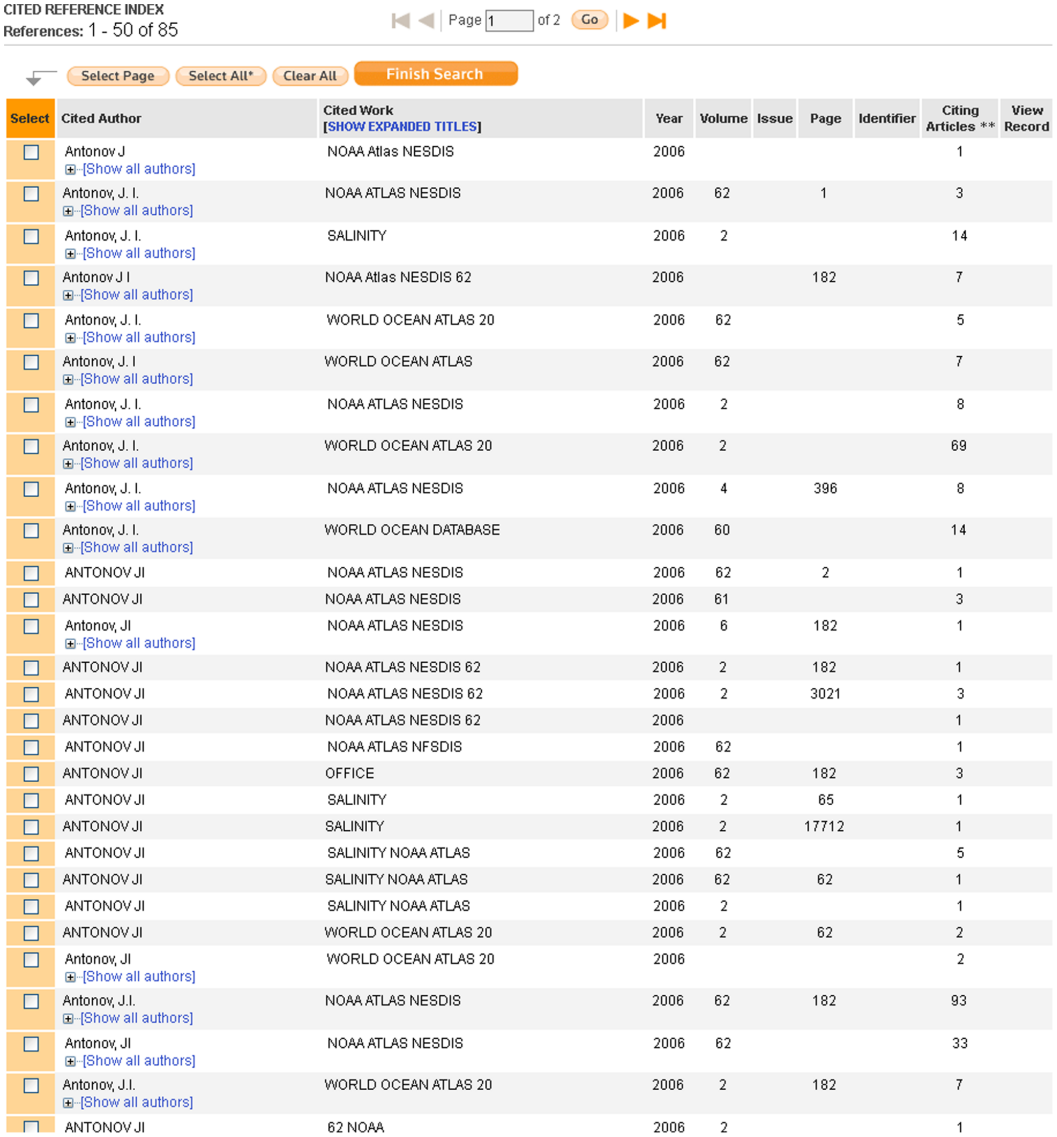
Variant methods of citing the 2005 version of the WOA/WOD in WoS.

Finally, as with phase 1 of this analysis, I find the format of citations to the WOA/WOD to be highly inconsistent. I found 377 variant methods of citing the 1982 version, 305 variants of the 1998 version, 221 variants of the 2001 version, 200 variants of the 2005 version, and 77 variants of the 2009 version, for a total of 1,180 variant methods of citing all versions of the WOA/WOD captured in WoS as of early 2013. See Figure 5 for a sample of the citation variants to the 2005 version. Because I did not attempt to search for erroneous citations (citations to the wrong year of publication, misspelled author names, etc), these figures are likely to underestimate the actual number of variant methods of citing the WOA/WOD in WoS. In addition, since phase 1 of this analysis suggests that articles are more likely to reference data sets in their text than in their cited references lists, the actual number of methods that articles use to refer to the WOA/WOD is likely to be substantially higher than I estimate here.

## Discussion

These results seem to have a number of implications for data curation and data citation initiatives. First, my results indicate that all three of these data sets are highly cited. My phase 1 results suggest that, if they were counted as journal articles in WoS, both the WOA/WOD and the PSST data sets would have citation counts higher than 99% of all articles in Oceanography in WoS from any single publication year from 1995 to the present. Using the more expansive journal full-text method, each of the three data sets would be ranked in the top 1% for citation counts of all articles published in Oceanography during the same year, while the WOA/WOD and PSST data sets would be ranked in the top 0.1%. My phase 2 results indicate that each version of the WOA/WOD would be ranked in the top 0.1% of articles in Oceanography that were published during the same year and the 1982 and 1994 versions have been cited more than twice as often as the most highly cited article in Oceanography published in 1982 and 1994. Percentile values and article citation counts for journal articles in Oceanography were obtained by using the search string “WC = oceanography AND PY = 1995” and sorting the results by “Times Cited – highest to lowest.” This string was then repeated for the other publication years. Because of the limitations of my search methods noted above, these citation counts are likely to be underestimates of the actual totals for each data set.

These high citation counts are surprising in light of the fact that previous studies [24], [52]–[54] have reported more modest citation counts to individual data sets. I speculate that the high citation counts reported here could result from the unique features of the particular data sets that I analyzed. First, each data set is freely and publically available and has been described in enough detail to permit its reuse. Second, the GHRSST and WOA/WOD data sets are composites of multiple smaller data sets contributed by multiple researchers to form a more comprehensive, global data set. Third, each data set has been available from a consistent source for an extended period of time. Finally, each data set is quality controlled to ensure the consistency and accuracy of the data contained in each set. Each of these features adds value to the original data sets, making the final data sets more useful to the oceanographic community. It may be that the high citation counts to these data sets, and particularly to the WOA/WOD, reflect the somewhat unique nature of these data sets.

If this is the case, it suggests a potential path forward for data repositories and data sharing in other disciplines. A single data set in isolation may have limited applications because of the methodology and parameters of its collection, but if that data set is quality controlled, adjusted, and merged with other similar data sets, it can be used to create a more comprehensive, overarching data product that can be queried for analysis at local, regional, or global disciplinary scales. The more data incorporated into such a product, the more useful the product becomes. Data repositories creating such products could then become central hubs for disciplinary, and potentially interdisciplinary, research, leveraging the limited research funding available in each discipline to ensure that individual pieces of research performed in that discipline eventually benefits the entire disciplinary community. In a sense, such a model could be considered a quality-controlled Wikipedia of data – the combination of individual pieces of expertise to create a resource larger and more comprehensive than anything that could be achieved individually. Obviously there are significant social, technological, and political barriers to implementing such a model, but the examples in oceanography of NODC and the Intergovernmental Oceanographic Commission [73] show that such barriers can be overcome.

Second, my results suggest that the majority of references to these data sets occur in the full text of articles, rather than in the title, abstract, keywords, acknowledgements, or cited references sections of these articles. The citation counts retrieved from full text sources—publishers' websites and Google Scholar—are consistently and substantially higher than those retrieved from WoS. The only exception is that the publishers' website total for WOA/WOD is lower than my phase 2 results, but this may be due to the large number of reference variants I found during phase 2. This pattern suggests that most articles do not refer to these data sets in a section indexed by WoS, calling into question the appropriateness of citation-indexing databases for compiling citation counts for these data sets.

Third, I find wide disparities in the methods used to cite or refer to these data sets, despite the fact that a formal citation format is suggested for each. This suggests that although a suggested citation format exists, researchers are not, for whatever reason, using it consistently to refer to these data sets. This finding is consistent with that of Part 2 of Mooney and Newton [28] and with many anecdotal accounts of citation practices among authors. It is likely that the multiple points of access to these data sets may account for some of this inconsistency. PSST, for example, is also available from NASA, leading some to refer to the data set as the ‘NASA Pathfinder SST’ data set. The implication of this trend for the data citation community seems to be that although the development of a standard citation format is necessary, that format by itself is not sufficient to guarantee consistent citation of data sets. It seems that in addition to developing this format, it will be necessary to encourage researchers to use the format and, perhaps more importantly, to obtain commitments from journal editors, reviewers, and publishers to ensure that it is used.

More consistent adoption and usage of a doi to refer to a data set, either by directly assigning a doi to a data set or through the publication of a data paper with a doi, has the potential to considerably reduce the issues resulting from this inconsistency. From a purely bibliometric perspective, the format and content of a reference or citation is irrelevant as long as a doi is present. The consistent use of a doi to refer to a data set would enable a researcher to search full-text or citation-indexing databases for that doi to retrieve a reasonably accurate set of articles citing that data set. Again, however, such consistency requires both data providers to assign dois to their data sets and authors to include these dois in their papers. NODC has begun to lay the groundwork for such consistency in oceanography, having recently assigned its first doi to a version of the PSST data set and developing a process for assigning dois to its other data sets.

## Conclusion

In this analysis, I attempted to generate citation counts for three oceanographic data sets curated by NODC by searching WoS, publishers' websites, and Google Scholar for mentions of these data sets in the bibliographic information or full text of scientific articles. I found that although there were substantial differences in the citation counts derived from each source, all three data sets were highly cited in all sources. The WOA/WOD was particularly highly cited, with all versions of the data set having received over 8,000 citations since its first release in 1982. My results suggest that scientific articles are more likely to mention these data sets in the text than in the acknowledgements or cited references sections. I also find wide discrepancies in the methods used to refer to these data sets, both in the full text and in the cited references sections. I found 377 variant methods of citing different versions of a single data set, WOA/WOD, suggesting that researchers are not consistently using the citation formats provided for these data sets.

Although I limited this analysis to oceanographic data sets in an attempt to control for potential differences in citation practices among fields [74], it seems likely that the findings and issues identified here may be similar for data sets in other disciplines. Previous studies have suggested that data sets in other disciplines are highly cited [54], [56] and that references to data sets retrieved from full text sources are higher than formal citation counts [24], both of which are consistent with the findings presented here. In addition, inconsistent referral to data sets in scientific papers is often raised as a motivator for the development of a data citation standard, suggesting that the large number of reference variants identified here is likely to be an issue with data sets in other disciplines as well.

However, although the findings of this analysis may be broadly applicable to data sets in other disciplines, much additional research would be needed to determine what, if any, differences exist in data citation and referencing patterns among disciplines. Since it is known that citation practices for journal articles differ among disciplines [74], it is likely that such differences also exist for data sets. Such differences may be compounded by differences in disciplinary collaboration rates, the existence and utilization of discipline-specific data repositories, or other factors.

In addition to citation counts, future research on the impact of data sets and data curation activities might focus on alternative metrics such as download counts, social network discussion, or social bookmarking to measure other forms of engagement with these data sets beyond formal citation [13], [75], or on comparing such altmetric indicators with traditional cited reference counts. Although download counts could be easily obtained, other altmetric indicators might be more problematic to obtain due to the inconsistency with which data sets are cited. Unique identifiers such as dois might alleviate this issue somewhat, but only for data sets that have been assigned such identifiers and to the degree that researchers include these identifiers in their publications.

Finally, this analysis demonstrates that individuals and institutions can make substantial contributions to scientific research without producing formal publications. My results suggest that these data sets are often used in the production of original research in oceanography. This use is possible because researchers posted their data sets to oceanographic data repositories and because these data sets were properly archived, described, and made available to the scientific research community. The high citation counts identified here suggest that these data sets – and, by extension, the curation activities necessary for their use in scientific articles – are at least as important to the advancement of oceanographic research as the findings presented in the vast majority of journal articles published in the field. Future evaluations of NODC and other organizations that curate scientific data ought to take such considerations into account.
